# The association between chronic venous disease and measures of physical performance in older people: a population-based study

**DOI:** 10.1186/s12877-021-02528-9

**Published:** 2021-10-14

**Authors:** Suvi-Päivikki Sinikumpu, Maija-Helena Keränen, Jari Jokelainen, Sirkka Keinänen-Kiukaanniemi, Laura Huilaja

**Affiliations:** 1 Department of Dermatology, University Hospital of Oulu, Oulu, Finland and Medical Research Center, PEDEGO Research Group, University of Oulu, P.B.20, OYS, FIN-90029 Oulu, Finland; 2 Medical Research Center, Clinical Neuroscience Research Group, University of Oulu, Oulu, Finland; Department of Geriatrics, Oulu University Hospital of Oulu, Oulu, Finland; 3 Northern Finland Birth Cohorts, Artic Biobank, Infrastructure for Population Studies, Faculty of Medicine, University of Oulu, Finland;, Oulu University Hospital, Oulu, Finland; 4 Center for Life Course Health Research, Faculty of Medicine, University of Oulu, Oulu, Finland; 5 Healthcare and Social Services of Selänne, Pyhäjärvi, Finland

**Keywords:** Older people, Chronic venous disease, Short Physical Performance Battery (SPPB), 10 m walk test, Leg ulcer

## Abstract

**Background:**

Muscle pump dysfunction is an essential component of chronic venous disease (CVD) pathology. Aging reduces muscle strength which further weakens the venous return. However, the epidemiology of CVD and its relationship with the physical performance in older persons is poorly studied. We studied the prevalence of CVD in subjects aged over 70 years and its association primarily with the Short Physical Performance Battery (SPPB) and 10 m walk test.

**Methods:**

An accurate clinical leg examination was performed and the Clinical-Etiological-Anatomical-Pathophysiological-classification (CEAP, clinical classification of chronic venous disorders, C1-C6) determined by dermatologists in 552 subjects aged between 70 and 93 years belonging to the Northern Finland Birth Cohort 1966 – Parents’ Study (NFBC-PS). Linear regression analyses were used to examine the association between CVD and functional tests and anthropometric measurements.

**Results:**

The prevalence of CVD (C1-C6) was 54.3%. C1 was diagnosed in 22.1% (n=84), C2 in 15.2% (n=45), C3 in 8.2% (n=45), C4 in 7.8% (43), C5 in 0.4% (n=2) and C6 in 0.5% (n=3). The prevalence and severity of CVD increased with increasing age (p<0.05). Males presented more with severe stages of CVD (C4-C6) (p<0.001). Subjects with CVD had significantly lower total SPPB scores and longer times in the 10 m walk test (p<0.001). The association between CVD severity and SPPB remained statistically significant in females after adjusting for age, body mass index (BMI) and number of children. The 10 m walk test times were associated with CVD when adjusted for sex and age but not after adjusting for BMI.

**Conclusions:**

It is recommended that detailed skin examination of legs should be performed by physicians treating older subjects in order to improve early diagnosis of CVD. We highlight the importance of physical activity in older persons - lower limb activation of older persons with CVD may improve venous return and therefore prevent progression of CVD. We found an association between CVD and gait speed, however, there may exist bidirectional relationship.

**Supplementary Information:**

The online version contains supplementary material available at 10.1186/s12877-021-02528-9.

## Background

Chronic venous disease (CVD), including varicose veins and chronic venous insufficiency (CVI), is a common medical condition in adults [1–3]. CVD affects people globally, but is most prevalent in developed countries [2, 4, 5]. The most severe stage of CVD is venous leg ulcer [1]. CVD has various consequences both for the individual and society. Venous diseases impact a person’s quality of life (QoL) causing symptoms like pain, weight sensation, itching and diminished mobility [6]. Moreover, investigations of CVD, wound care and hospitalization impose a substantial financial burden on society [7, 8]. The main etiology of CVD is chronic venous hypertension and venous reflux that develop after the calf-muscle pump dysfunction [9]. In older persons, muscle strength is reduced which then weakens the venous return in valves [10]. In addition, physical activity has other undisputed health benefits in older people: it improves a quality of life, reduces disability, mortality and prevents for chronic diseases [11, 12]. In turn, lower walking speed, leg strength and balance are associated with higher risk of mortality in persons over 70 [13]. However, regardless of multiple benefits of physical activity older adults are sedentary and have low level of physical activity, especially women and older age groups [11].

Approximations of CVD occurrence differ greatly in study methods, diagnostic criteria, geographic regions, selection of study subjects and ethnicity [1–5, 7, 14–19]. However, subjects in previous studies were moderately young and epidemiological studies among older persons are lacking [4, 8, 20, 21]. A recent international survey (The Vein Consult Program), with over 90 000 subjects in 23 countries, found clinically significant CVD in approximately 70% of the participants (mean age 51.8 years) [4] and reported an increase in predicted probability of CVD with age, being highest in subjects aged over 65 years. Data in that study was collected from the population visiting general practitioners for various reasons. A similar finding was seen in the population-based Bonn Vein Study (n=3072, aged 18 to 79 years) in which the prevalence of CVD was highest in subjects aged 60 to 80 years [15]. In the pioneering population-based Edinburgh Vein Study (n=1566, ages 18 to 64 years) the prevalence of varicose veins was 55.7% in those aged 55 to 64 years being significantly lower in those aged from 35 to 44 years (28.8%) [20].

Due to aging of the Western population, the prevalence of CVD is projected to increase in the coming years [2]. Thus, it is alarming that, despite international guidelines, CVD is poorly recognized by physicians [22–24]. There is an urgent need for improved awareness of CVD and its risk factors. Moreover, it is important to highlight the importance of physical activity in older persons in means to improve muscle strength and further prevent morbidity. The primary aim of this study was to determine the prevalence of CVD in subjects aged over 70 from the Northern Finland Birth Cohort 1966 - Parents’ Study (NFBC-PS) by using the Clinical-Etiological-Anatomical-Pathophysiological classification (CEAP, C0-C6) [24]. Secondly, we wanted to study the association between CVD and Short Physical Performance Battery (SPPB) and 10 m walk test performance since power in lower extremity muscles is known to reduce with aging and thus weakening of the venous return. In addition, the relationship between CVD and body mass index (BMI), body composition, blood pressure, the number of children (females), socioeconomic status (SES), sex and living status in the older population was examined.

## Methods

### Study cohort, dermatological examination and CEAP classification

The Northern Finland Birth Cohort 1966 (NFBC1966) is an epidemiological and longitudinal research program in the two northernmost provinces in Finland (Oulu and Lapland). The NFBC1966 comprised the offspring of the mothers, who lived in either province and whose expected delivery date fell between 1st January and 31st December 1966 [25]. The surviving parents of the subjects in the NFBC1966 have participated in a comprehensive health study called NFBC-PS and invited to take part in a diverse health questionnaire (Keränen et al., unpublished observations) [26]. All participants living in the Oulu area (n=1239) were also invited to participate in the clinical examination, including total body skin examination.

A skin examination was performed on in 552 participants (346 females and 206 males) and this subpopulation was included in the final skin study analysis. The skin data was collected between May 2018 and March 2019 on the premises of the Faculty of Medicine of the University of Oulu [26].

**Fig 1:**
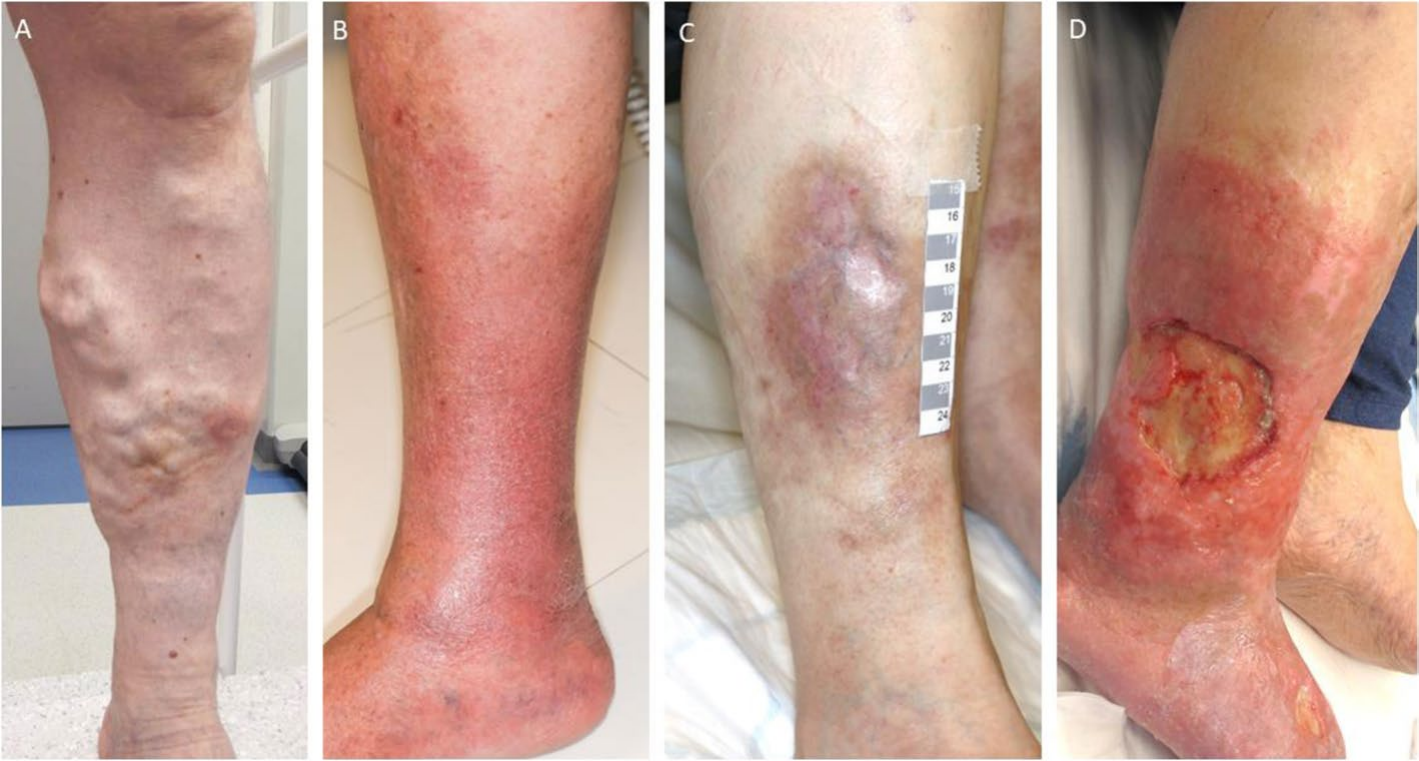
Skin findings in chronic venous disease (clinical CEAP classification in parenthesis). **A** edema and varicose veins (C3) **B** stasis dermatitis (C4) **C** healed venous ulcer, post-inflammatory pigmentation and lipodermatosclerosis (C5) **D** active venous ulcer (C6).

The comprehensive clinical survey included a thorough 20-minute whole-skin examination performed by experienced dermatologist. All areas of the skin were observed including the nails, hair and scalp, with a detailed inspection of the legs. CVD was categorized using the clinical CEAP classification [24]: C0 No visible or palpable signs of venous disease, C1 telangiectasias or reticular veins, C2 varicose veins (distinguished from reticular veins by a diameter of 3 mm or more), C3 edema, C4 changes in skin and subcutaneous tissue secondary to CVD, pigmentation or eczema, lipodermatosclerosis or atrophie blanche, C5 healed venous ulcer and C6 active venous ulcer (Fig 1). Stages C1-C3 were categorized as mild or moderate disease and stages C4-C6 as severe disease (CVI refers to an advanced form of CVD with skin changes and ulceration [CEAP, severe diseases C4-C6]). During inspection of the legs, subjects were standing on a platform with their feet in three standard positions: facing the examiner with heels together and feet spread wide apart, facing away from the examiner in a similar position, and facing away from the examiner with feet parallel as in the Edinburgh Vein Study [20]. Subjects remained in a standing position for at least two minutes before examination of their veins, to allow blood to pool in the legs. All signs of varicose veins and skin findings were recorded.

### The Short Physical Performance Battery (SPPB), 10 m walk test and anthropometric measurements

The Short Physical Performance Battery (SPPB) included the chair stand, gait speed and balance tests [27]. The SPPB was calculated according to literature standards [27]. The total score of SPPB ranged from 0 to 12 while every sub-test ranged from 0 (worst performance) to 4 (best performance). The study nurse first demonstrated each test for participants. To test the ability to stand up from the chair, study members were asked to hold their arms on their chest, rise from the chair and sit down again five times as quickly as possible. The balance test started with a semi tandem position, in which the heel of one foot was placed to the side of the first toe of the other foot with the participant choosing which foot to place forward. The timing was stopped when the study member moved their feet or when 10 seconds was elapsed, whichever was sooner. Those who were able to hold a semi tandem position were further evaluated by a balance test in tandem position. Those unable to hold a semi tandem position were asked to do a test with the feet in a side by side position. In the gait speed test, participants were instructed to walk 4 m in a spacious corridor. Distances were provided at the beginning and end of the timed walkway to allow participants space to accelerate/decelerate outside the data collection area. Participants were instructed to “walk at your comfortable, usual pace” until they reached the end of the marked path. The time taken, in seconds, for the 4 m walk test was recorded. The 10 m walk test followed the same procedure.

The clinical examination included body weight in light clothing, measured with a digital scale. Height was recorded as the mean of two measurements using a standard calibrated stadiometer. Body mass index (BMI) was calculated as the ratio of weight to height squared (kg/m^2^). Body composition was analyzed by multifrequency bioelectric impedance analysis (BIA; InBody960, Biospace, Seoul, Korea). Study participants were classified into five groups, based on their BMI, according to WHO criteria: underweight (<18.5), normal (18.5–25), overweight (25–30), obese (30–35) and severely obese (>35). Systolic and diastolic blood pressure was measured three times a minute apart after 15 minutes of rest on the right arm of seated participants using an automated oscillometric blood pressure device and appropriately sized cuff (Omron Digital Automatic Blood Pressure Monitor Model M10-IT, Japan). The mean of the three systolic and diastolic values was used in the analyses. Test results were recorded by authorized study nurses.

### Self-reported questionnaires and medical history

The SES of the study subjects was defined as educational level [28]. The study cases were classified into three subgroups according to their highest level of education: 1) primary school, 2) secondary school and 3) post-secondary level education/vocational college/university. Information on education, living status (alone or with a spouse/other family member), and possible risk factors for CVD (the number of children and comorbidities; history of congestive heart failure, atherosclerosis of native arteries of the extremities, diseases of the musculoskeletal system and connective tissue) were self-reported in health questionnaires. Patient records (history of previous deep venous thrombosis, previous interventional management of CVD and hip fraction) were obtained from the Finnish Care Register for Health Care, maintained by the National Institute of Health and Welfare, and selected by diagnoses based on the International Classification of Diseases (ICD-10 codes I80.0, I80.1, I80.20, I80.29, I80.3, I80.8, I80.9, S72.0 and intervention codes PHB76-78, PHD78, PHM76-79, PHN75, PHS78, PHT99, PHW96, TPH10, PH2AC, PH2AE, PH2BC) (Supplementary data).

### Statistical methods

Chi-squared test, T-test or ANOVA were used to describe the categorical and continuous characteristics of the study sample, respectively. Linear regression analyses adjusted for age, gender and BMI were performed to study associations of CVD severity (mild C1-C3 or severe C4-C6) with the SPPB score and time spent on the 10 m speed test as dependent variable. In addition, for females the analyses were adjusted for the number of children. Regarding to history of self-reported diseases (the history of congestive heart failure, atherosclerosis of native arteries of the extremities and diseases of the musculoskeletal system and connective tissue) there was not statistical difference (p-value>0.05) between those without CVD (C0) and with CVD (C1-C6) which is the reason these were not included to the adjusted model. The linearity assumption was checked by inspecting the Residuals vs Fitted plot, and the Q-Q plot of residuals was used to visually check the normality assumption. The interaction between SPPB CVD and sex was assessed, and models were made separately for males and females. P-values <0.05 were considered statistically significant. Analyses were performed using R, version 4.0.2 (https://www.R-project.org/).

### Ethical approval

The Ethical Committee of the Northern Ostrobothnia Hospital District approved the study (115/2012) which was performed according to the principles of the Helsinki Declaration of 1983.

## Results

The mean (SD) age at the time of the skin study was 78.4 (4.18), with a range of 70–93 years. Subject demographics are presented in Table 1.

**Table 1:** Demographics of study participants (n/%, unless other stated)

	All^**a**^ ***n***=552	Males ***n***=206	Females ***n***=346	***p***-value
Sex^1^				<0.001
Men	206 (37.3%)	206 (100%)	0 (0.00%)	
Women	346 (62.7%)	0 (0.00%)	346 (100%)	
Ethnicity (white)	552 (100.0%)	206 (100%)	346 (100%)	<0.001
Living status^1^				<0.001
Living alone	164 (29.9%)	29 (14.1%)	135 (39.2%)	
Living with spouse or other family member	385 (70.1%)	176 (85.9%)	209 (60.8%)	
Education^1^				0.105
No education/primary school	104 (21.4%)	31 (16.5%)	73 (24.4%)	
Secondary school	193 (39.6%)	77 (41.0%)	116 (38.8%)	
Post-secondary level education/vocational college/university	190 (39.0%)	80 (42.6%)	110 (36.8%)	
Age (years), mean (SD)^2^	78.4 (4.18)	79.2 (3.71)	78.0 (4.38)	0.001
Age ranges^1^				0.001
70-75	108 (19.6%)	22 (10.7%)	86 (24.9%)	
75-80	234 (42.4%)	95 (46.1%)	139 (40.2%)	
80-85	162 (29.3%)	71 (34.5%)	91 (26.3%)	
85-90	43 (7.79%)	16 (7.77%)	27 (7.80%)	
90-93	5 (0.91%)	2 (0.97%)	3 (0.87%)	
Systolic blood pressure, (mmHg), mean (SD)^2^	142 (21.3)	142 (21.7)	142 (21.1)	0.757
Diastolic blood pressure, (mmHg), mean (SD)2	82.7 (11.2)	80.6 (10.9)	83.9 (11.2)	0.001
Weight (kg), mean (SD)^2^	72.3 (13.1)	80.2 (13.1)	68.0 (11.0)	<0.001
Body mass index, BMI (kg/m^2^), mean (SD)^2^	27.3 (4.28)	26.9 (3.63)	27.5 (4.61)	0.104
Number of children (females), mean	3.02 (2.01)	. (.)	3.02 (2.01)	.
Total SPPB score, mean (SD)^2^	10.0 (1.86)	10.3 (1.60)	9.87 (1.99)	0.011
10 m walk test, seconds, mean (SD)^2^	8.86 (2.08)	8.67 (1.98)	8.97 (2.13)	0.108
History of venous interventions^1^				0.330
No	491 (94.2%)	183 (95.8%)	308 (93.3%)	
Yes	30 (5.76%)	8 (4.19%)	22 (6.67%)	
History of deep venous thrombosis^1^				1.000
No	525 (97.4%)	192 (97.5%)	333 (97.4%)	
Yes	14(2.60%)	5 (2.54%)	9 (2.60%)	1.000
Hip fraction^1^	7(1.27%)	4(1.94%)	3(0.87%)	0.433
Body composition measurements:^2^				
Body mass index (kg/m^2^), mean (SD)	27.0 (4.03)	26.9 (3.74)	27.1 (4.19)	0.689
Body fat mass (kg), mean (SD)	25.6 (8.64)	23.5 (8.84)	26.7 (8.33)	0.001
Fat free mass (kg), mean (SD)	46.7 (9.22)	56.8 (6.74)	41.2 (4.65)	<0.001
Percentage body fat (%),mean (SD)	35.0 (8.41)	28.5 (6.91)	38.5 (6.92)	<0.001
Skeletal muscle mass (kg), mean (SD)	25.0 (5.75)	31.1 (3.95)	22.0 (2.75)	**<0.001**
Percentage skeletal muscle mass (%), mean (SD)	34.8 (5.57)	39.2 (3.81)	32.7 (3.69)	**<0.001**
Muscle fat ratio, mean (SD)	111 (52.2)	150 (58.3)	90.2 (31.2)	**<0.001**

^a^There is some missing data because all did not answer to questionnaires or denied the use of data afterwards

1 Statistical analyses performed by Chi-Square test

2 Statistical analyses performed by T-test

According to the clinical examination the prevalence of CVD (CEAP C1-C6) was 54.3% in the sample. Table 2 summarizes the sex- and age- specific percentage distributions of CVD. Both the prevalence of CVD and the disease severity increased with age (p<0.01). CVD affected more females than males but males had a higher rate of severe disease (p<0.01). The demographic characteristics and physical performance test results between three groups divided by CEAP severity classification are shown in Table 2. SPPB scores showed a significant association with CEAP classification. The total score in SPPB (from 0 to 12) was significantly lower in those with CVD when compared with those with a C0 classification (p<0.001) and the total score decreased with increased CVD severity. There was no significant difference in the grade of CVD between groups based on the 4 m speed test whereas a difference was demonstrated in the 10 m speed test (p<0.05). Those with CVD had also more venous interventions and venous thrombosis in their history than those without CVD (C0) (p<0.05). Subjects with CVD presented with higher weight, height and BMI. CVD severity increased by the increasing body fat mass (p<0.01, Table 2). In gender specified analyses, this was seen only in males (p<0.01, Table 3). Higher skeletal muscle mass associated with the CVD severity in males (C0 vs C1-C6, p<0.01) (Table 3).

**Table 2:** Clinical CEAP classification by subject demographics and functional tests

	C0^**a**^ ***N***=252	C1-C3^**a**^ ***N***=251	C4-C6^**a**^ ***N***=48	***p***-value
Sex^1^				<0.001
Female	138 (40.1%)	180 (52.3%)	26 (7.56%)	
Male	114 (55.6%)	69 (33.7%)	22 (10.7%)	
Living status^1^				0.064
Living alone	65 (39.6%)	87 (53.0%)	12 (7.32%)	
Living with spouse or other family member	186 (48.4%)	162 (42.2%)	36 (9.38%)	
Education^1^				0.248
No education/Primary school	42 (40.4%)	56 (53.8%)	6 (5.77%)	
Secondary school	95 (49.5%)	78 (40.6%)	19 (9.90%)	
Post-secondary level education/vocational college/university	89 (46.8%)	87 (45.8%)	14 (7.37%)	
Age, mean (SD)^2^	77.9 (4.08)	78.8 (4.11)	79.0 (4.66)	0.047
Age ranges				0.143
70–75	59 (54.6%)	42 (38.9%)	7 (6.48%)	
75–80	113 (48.3%)	103 (44.0%)	18 (7.69%)	
80–85	62 (38.3%)	84 (51.9%)	16 (9.88%)	
85–90	16 (38.1%)	19 (45.2%)	7 (16.7%)	
90–93	2 (40.0%)	3 (60.0%)	0 (0.00%)	
Systolic blood pressure, (mmHg), mean (SD)^2^	142 (22.0)	141 (20.6)	144 (22.2)	0.780
Diastolic blood pressure, (mmHg), mean (SD)^2^	82.6 (11.1)	82.8 (10.8)	83.0 (13.6)	0.962
Lenght, cm, mean (SD)^2^	164 (8.82)	163 (8.67)	166 (9.49)	0.032
Weight, kg, mean (SD)^2^	72.5 (12.3)	72.2 (13.3)	81.4 (16.4)	<0.001
Body mass index (kg/m^2^), mean^2^ (SD)	26.9 (3.77)	27.3 (4.48)	29.6 (5.05)	<0.001
Balance test, score, mean (SD)^2^	3.60 (0.82)	3.52 (0.84)	3.13 (1.10)	0.003
4 m walk test, score, mean (SD)^2^	3.94 (0.52)	3.91 (0.35)	3.86 (0.42)	0.496
Chair stand test,score, mean (Sd)^2^	2.74 (1.14)	2.67 (1.08)	2.24 (1.18)	0.020
SPPB, total score, mean (SD)^2^	10.2 (1.71)	10.0 (1.81)	9.00 (2.55)	<0.001
10 m walk test, seconds, mean (SD)^2^	8.63 (1.90)	8.97 (2.23)	9.44 (2.01)	0.031
Use of ancillary in SPPB or walk test^1^				0.007
No	241 (45.7%)	244 (46.3%)	42 (7.97%)	
Yes	6 (42.9%)	3 (21.4%)	5 (35.7%)	
Number of children (females)^1^	2.85 (1.53)	3.09 (2.12)	3.50 (3.11)	0.258
History of venous interventions^1^				0.018
No	234 (47.7%)	217 (44.2%)	40 (8.15%)	
Yes	8 (26.7%)	16 (53.3%)	6 (20.0%)	
History of deep venous thrombosis^1^				0.021
No	244 (46.5%)	238 (45.3%)	43 (8.19%)	
Yes	3 (21.4%)	7 (50.0%)	4 (28.6%)	
Hip fraction^1^	1(0.40%)	4 (1.59%)	2 (4.17%)	0.057
Body composition measurements: ^2^				
Body mass index (kg/m^2^), mean (SD)	26.7 (3.76)	27.0 (4.03)	29.2 (4.92)	0.007
Body fat mass (kg), mean (SD)	24.7 (8.09)	25.7 (8.54)	30.4 (10.9)	0.004
Fat free mass (kg), mean (SD)	47.4 (9.44)	45.5 (8.66)	49.8 (10.2)	0.028
Percentage body fat (%), mean (SD)	33.9 (8.05)	35.6 (8.58)	37.2 (9.01)	0.058
Skeletal muscle mass (kg), mean (SD)	25.2 (6.23)	24.5 (5.12)	26.9 (5.91)	0.096
Percentage skeletal muscle mass (%), mean (SD)	35.1 (6.21)	34.6 (4.91)	33.8 (5.08)	0.395
Muscle fat ratio, mean (SD)	114 (47.3)	109 (56.8)	102 (53.5)	0.479

C0; no CVD disease, C1-C3 mild CVD, C4-C6 severe disease. Based on the CEAP, clinical classification of chronic venous disorders

^a^There is some missing data because all did not participate to all measurements or did not answer to questionnaires or denied the use of data afterwards

*CEAP* Clinical-Etiological-Anatomical-Pathophysiological, *SPPB* Short Physical Performance Test

1 Statistical analyses performed by Chi-Square test

2 Statistical analyses performed by ANOVA

**Table 3:** Clinical CEAP classification by body composition measurements in both sex^a^

a) Males					
	Total^**b**^ ***N***=129	C0 ***N***=73	C1-C3 ***N***=43	C4-C6 ***N***=13	***p***-value
Body mass index (kg/m^2^), mean (SD)	26.9 (3.74)	26.5 (3.22)	26.6 (3.74)	30.0 (5.19)	0.007
Body fat mass (kg), mean (SD	23.5 (8.84)	22.5 (7.32)	22.7 (8.78)	31.5 (12.8)	0.002
Fat free mass (kg), mean (SD)	56.8 (6.74)	56.0 (6.70)	57.3 (6.51)	59.1 (7.56)	0.269
Percentage body fat (%), mean (SD)	28.5 (6.91)	28.1 (5.76)	27.6 (7.27)	33.6 (9.64)	0.015
Skeletal muscle mass (kg), mean (SD)	31.1 (3.95)	30.7 (3.94)	31.4 (3.86)	32.3 (4.28)	0.347
Percentage skeletal muscle mass (%), mean (SD)	39.2 (3.81)	39.4 (3.15)	39.7 (4.03)	36.2 (5.28)	0.011
Muscle fat ratio, mean (SD)	150 (58.3)	148 (43.6)	162 (73.6)	124 (68.0)	0.115
**b) Females**					
	**Total**^**b**^ ***N*=236**	**C0** ***N*=98**	**C1-C3** ***N*=122**	**C4-C6** ***N*=16**	***p*-value**
Body mass index (kg/m^2^), mean (SD)	27.1 (4.19)	26.8 (4.14)	27.1 (4.14)	28.6 (4.75)	0.270
Body fat mass (kg), mean (SD	26.7 (8.33)	26.3 (8.29)	26.7 (8.23)	29.5 (9.29)	0.357
Fat free mass (kg), mean (SD)	41.2 (4.65)	40.9 (4.94)	41.4 (4.53)	42.3 (3.70)	0.517
Percentage body fat (%), mean (SD)	38.5 (6.92)	38.3 (6.64)	38.5 (7.08)	40.2 (7.54)	0.603
Skeletal muscle mass (kg), mean (SD)	22.0 (2.75)	21.8 (2.94)	22.0 (2.68)	22.5 (2.10)	0.675
Percentage skeletal muscle mass (%), mean (SD)	32.7 (3.69)	32.9 (3.55)	32.7 (3.76)	31.8 (4.05)	0.538
Muscle fat ratio, mean (SD)	90.2 (31.2)	90.4 (27.2)	90.9 (34.3)	84.1 (29.5)	0.715

C0; no CVD disease, C1-C3 mild CVD, C4-C6 severe disease CVD. Based on the CEAP, clinical classification of chronic venous disorders ^a^Statistical analyses was performed by ANOVA

^b^There is some missing data because every study case did not participate to body composition measurement

As shown in Table 4, the association between SPPB and severe (C4-6) CVD remained statistically significant in females after adjusting for BMI, age and number of children (when compared with the group C0). In males the corresponding association disappeared after adjusting. In the 10 m speed test there was significant association between CVD after adjusting for sex and age (data not shown). However, the association disappeared after adjusting for BMI.

**Table 4:** Association between clinical CVD and SPPB or 10 m walk test

	***Dependent variable:***			
	SPPB^**a**^			10 m walk test^**b**^
	**Males (n=206)**	**Females (n=346)**		**All (n=552)**
	β (SE) ^c^	β (SE) ^c^		β (SE)
Chronic venous disease, stages C1-C3	-0.313 (0.236)	0.191 (0.217)		0.104 (0.184)
Chronic venous disease, stages C4-C6	-0.421 (0.377)	-1.178^***^(0.409)		0.422 (0.334)
Male				-0.358^**^(0.182)
Body mass index (mean)	-0.073^**^(0.032)	-0.107^***^ (0.024)		0.119^***^(0.022)
The number of children		-0.041 (0.051)		

C0; no CVD disease, C1-C3 mild CVD, C4-C6 severe disease CVD. Based on the CEAP, clinical classification of chronic venous disorders

^*^p<0.1; ^**^p<0.05; ^***^p<0.01

Linear regression model

^a^Adjusted with BMI, age and the number of children (females)

^b^Adjusted with age, sex and BMI

^c^ β (SE); Regressio coefficient

## Discussion

Our clinical and cross-sectional study is the first to determine the prevalence of CVD in a pure aged population by using the clinical CEAP classification. Results from this study confirm that CVD is common in older individuals, affecting every other person aged over 70 years. In addition, we found that severe types of CVD became more common with increasing age. CVD was more prevalent in females when compared with males whereas the most severe stages of CVD were predominantly seen in males. To best of our knowledge, this is the first study in which the association between physical performance tests (SPPB and 10 walk test) and CVD was researched. In these analyses we found that functional limitations in older persons were clearly associated with CVD and its severity.

Other studies showing that the prevalence of CVD increases with age are in line with our study [1–4, 15–19, 21, 29]. Nevertheless, previous studies have had some limitations: subjects have been rather young, or data on the age-specific prevalence of CVD have been incomplete and thus the real prevalence of CVD in the older population has stayed largely unknown. In addition, diagnostic criteria have varied between studies which makes comparison with the present study difficult. The Edinburgh Vein Study used a modified CEAP in diagnosing CVD and reported a 25% and 12% prevalence of CVI in male and female subjects, respectively, in patients aged 55 to 64 years [18]. The Vein Consult Program used solely the C-classification without ultrasound in diagnosis, corresponding with our study method. This large study reported the prevalence of CVI to be 26% (the mean age of the study population was 51.8 years) [4]. By comparison, the prevalence of CVI (CVD stages 4-6) was 45% in the current study, an observation most likely explained by the higher mean age (71.8 years) in our study.

Elderly subjects have many risk factors for CVD: multimorbidity, immobility, poor nutrition and weakened healing processes all increase the risk of varicose veins, skin changes and other venous alterations which further predisposes individuals for chronic leg ulcers [30]. Thus it is understandable that not only the prevalence but also the severity of CVD increases with age, as also seen in the present study [1, 4]. The socioeconomic impact of CVD in older subjects is substantial because of reduced quality of life. Heaviness of legs, itching, a feeling of swelling and cramps are common symptoms in CVD patients and complicate daily activities.

Venous ulcers are the most unwanted endpoint of CVD. Ulcers are often painful and keep sufferers from engaging in social activities, leading, in the worst cases, to depression [2]. Quality of life in patients with venous ulceration has been reported to be poor and comparable to that of patients with congestive heart failure or chronic lung disease [2]. CVD also imposes a significant burden on health care systems. Currently CVD consumes 2% of health care budgets in the US [2]. With an ageing population, it is unavoidable that the number of serious CVD events is going to increase.

In this study, the association between physical performance and CVD was surveyed by using the SPPB and the 10 m walk test. SPPB is a comprehensive and objective tool to evaluate lower extremity physical performance status and functional capability [27]. The SPPB has been selected from several observational studies and it has suggested to be a highly sensitive indicator of health status and an indicator of vulnerability thus predicting also all-cause mortality [27, 31]. In addition, the SPPB is easy to perform [31]. Similarly, walking speed (the 10 m walk test used in this study) is a widely used objective measure of functional mobility; it has been used to measure the association between health-related outcomes, disability, falls, hospitalization and mortality [32]. Interestingly we found that the total score of SPPB was significantly lower in those with CVD when compared with those with C0. Moreover, the total SPPB score decreased with increasing severity of CVD. Similarly, the time taken for the 10 m walk test increased with CVD severity. An essential component of venous pathology – besides hypertension and valvular incompetence – is muscle pump dysfunction, as demonstrated in several studies of patients with CVI [10, 33–35]. During the aging process muscle mass declines because of atrophy in type II muscle fibers [36]. Weakened calf and thigh muscle mass worsens venous return [10, 37]. Previously, the relationship between lower-extremity muscle strength and CVD has only been researched in a few studies with rather small sample sizes [10, 38–40]. All these studies demonstrated that calf muscle endurance is weakened in those with CVI. However, more future research with larger sample sizes and relevant clinical outcome measures has been called for [38]. As far as we know, this is the first study where the association between SPPB and CVD or its severity has been studied.

Varicose veins (C1-C2) affect more females when compared with males [41–43]. A higher number of full term pregnancies is a significant risk factor for varicose veins [44], partly explaining the difference. It has also been speculated that sex steroid hormone concentrations could play a role in the development of varicose veins [45] but in turn, the use of oral contraceptives or hormone replacement therapy have not showed undisputed risk for varicose veins [46]. We studied also the association between body composition and CVD separately in both sex. We found that in males those without CVD (C0) had higher skeletal muscle mass than those with CVD (C1-C6). In males the increasing severity of CVD was associated with the increasing body fat mass too. Correspondenly, females without CVD presented with higher percentage skeletal muscle mass when compared with those with CVD but the difference did not reach statistical significance. However, there is no consistent understanding as to whether the more severe stage of CVD are more common in females or males [15, 19, 47]. In the present study we found that, overall, female subjects presented more frequently with CVD but that the severe stages were more prevalent in males. When other risk factors of CVD were taken into account we demonstrated that CVD was associated with a history of venous interventions and venous thrombosis. Similarly the increasing number of children had relationship with the CVD severity, although the result was not statistically significant.

The major strengths of this study are twofold: a pure general population of older people and a moderately good sample size. Our study adds significantly to the knowledge of CVD in aged individuals, which has been much-needed. The clinical CEAP classification was used in this study as recommended by clinical practice guidelines [3]. The clinical examination of legs was performed by experienced dermatologists who are best qualified to differentiate the dermatological findings of CVD from other dermatological diseases. In addition, interobserver reliability was tested between the two main researchers (SPS, LH) and its degree was high [48]. The SPPB and 10 m walk test were chosen as an indicator of muscle strength as they measure functional limitations in older persons and have been used to identify people at greater risk for mortality and disability. These functional tests were performed by trained nurses instead of self-reporting information. The use of objective measures like the SPPB are preferred in older people because self-reporting is vulnerable to inherent bias and inaccuracy [49]*.* As a limitation of the study we acknowledge that no duplex ultrasound was performed to verify clinical diagnoses of CVD. However, the clinical CEAP is an inexpensive method, easy to do and available to all physicians. In addition, not all of the study cases invited chose to participate which may have led to selection bias.

## Conclusions

As a conclusion, we highlight the importance of dermatological evaluation of the lower extremities for older people. Skin examination enables the early identification of CVD and enhances the prospects of optimal treatment. Older subjects with clinical signs of CVD should be evaluated to assess their need for further investigations [50, 51]. They should be instructed about the use of compression bands whenever possible and proper foot care as means to diminish symptoms and prevent the progression of chronic ulcers. In addition, while physical activity has its evident benefits for health we recommend lower limb activation for older persons with CVD because it might improve the venous return and therefore prevent the progression of CVD.

## Supplementary Information

**Additional file 1:.**

## Data Availability

The data that support the findings of this study are available from Northern Finland Birth Cohort 1966 Study. Restrictions apply to the availability of these data, which were used under license for this study. Data are available at http://www.oulu.fi/nfbc/node/44315 with the permission of Northern Finland Birth Cohort.

## Abbreviations

CVD: Chronic venous disease
CVI: Chronic venous insufficiency
SPPB: Short Physical Performance Battery
CEAP: Clinical-Etiological-Anatomical-Pathophysiological classification
NFBC-PS: The Northern Finland Birth Cohort 1966 - Parents’ Study
NFBC66: The Northern Finland Birth Cohort 1966
