# Supplementary material for: The association between chronic venous disease and measures of physical performance in older people: a population-based study
Source: BMC Geriatr. 2021 Oct 14;21:556. doi: 10.1186/s12877-021-02528-9 (PMC8518156; doi:10.1186/s12877-021-02528-9)
Supplement: Supplementary file 1 — Additional file 1:. [file 12877_2021_2528_MOESM1_ESM.docx]

Supplemental data. Patient records (history of previous deep venous thrombosis and previous interventional management of CVD) were obtained from the Finnish Care Register for Health Care, maintained by the National Institute of Health and Welfare, and selected by diagnoses based on the International Classification of Diseases:

PHB76 Ligature of long saphenous vein

PHB77 Ligature of short saphenous vein

PHB78  Ligature of perforating veins of lower extremity

PHD78 Resection of communicating veins of lower leg and thigh

PHM76 Endovenous obliteration of v.saphena magna

PHM77 Endovenous obliteration of v.saphena parva

PHM78 Endovenous obliteration of perforating veins of lower extremity

PHM79 Endovenous obliteration of v.saphena anterio

PHN75 Plastic repair of popliteal vein

PHS78 Endoscopic interruption of perforating vein of lower leg

PHT99 Injection of therapeutic or sclerosing agent into other vein

PHW96 Other operation for vein insufficiens on lower leg veins

TPH10 Sclerotherapy of varicose veins

PH2AC  Flebography of lower extremity with X-ray

PH2AE Ultrasound examination of lower leg veins

PH2BC Extensive flebography of lower extremity with X-ray

I80.0 Phlebitis/thrombophlebitis venarum superficialium membrorum inferiorum

I80.1 Phlebitis/thrombophlebitis venae femoralis

I80.2 Phlebitis/thrombophlebitis venae iliacae [externae/internae/communis]

I80.29 Phlebitis/thrombophlebitis venarum profundarum membrorum inferiorum

I80.3 Phlebitis sive thrombophlebitis membri inferioris non specificata

I80.8 Phlebitis/thrombophlebitis aliis locis specificatis

I80.9 Phlebitis/thrombophlebitis loco non specificato

S72.0 Hip fraction
